# Identification of novel *MYO19* variants in neonatal hypertrophic cardiomyopathy: a familial analysis revealing oligogenic contributors to disease severity

**DOI:** 10.1186/s13023-025-03871-5

**Published:** 2025-07-09

**Authors:** Hye-Won Cho, Hyeseon Kim, Jeong-Min Kim, Dong Mun Shin, Oc-Hee Kim, Misun Yang, Heui Seung Jo, Mi-Ae Jang, Ja-Hyun Jang, Hyun-Young Park, Yun Sil Chang, Mi-Hyun Park

**Affiliations:** 1 Division of Genome Science, Department of Precision Medicine, National Institute of Health, Cheongju, Chungbuk 28159 Korea; 2 Department of Pediatrics, Samsung Medical Center, Sungkyunkwan University School of Medicine, 81 Irwon-Ro, Gangnam-Gu, Seoul, 06351 Korea; 3 Cell and Gene Therapy, Samsung Medical Center, Seoul, 06351 Korea; 4 Department of Pediatrics, Kangwon National University School Hospital, Kangwon National Univertisy School of Medicine, Chuncheon, 24289 Korea; 5 Department of Laboratory Medicine and Genetics, Samsung Medical Center, Sungkyunkwan University School of Medicine, Seoul, 06351 Korea; 6 National Institute of Health, Cheongju, 28159 Korea; 7 Department of Health Sciences and Technology, Samsung Advanced Institute for Health Sciences and Technology, Sungkyunkwan University, Seoul, 06351 Korea

**Keywords:** Hypertrophic cardiomyopathy, Neonate, Mitochondrial gene, *MYO19*, Compound heterozygous variant, Oligogenic inheritance

## Abstract

**Background:**

Pediatric hypertrophic cardiomyopathy (HCM) is a rare condition, particularly in neonates, and is characterized by rapid and extensive myocardial hypertrophy, often leading to severe clinical outcomes. HCM can arise from variants in sarcomeric genes, which are essential for myocardial contractions, as well as non-sarcomeric gene variants. Although genetic modifiers and oligogenic inheritance have been implicated in congenital heart disease and cardiomyopathy, their complexity in HCM has not been fully elucidated, especially in familial cases with variable phenotypes. Hence, this study aims to investigate the genetic architecture in a family with a history of cardiac disease and neonatal HCM, focusing on oligogenic inheritance of non-sarcomeric variants.

**Methods:**

Clinical data and blood samples were collected for genetic analysis. Whole genome sequencing (WGS) and bioinformatic analyses identified compound heterozygous variants in the *MYO19* gene. Maternally inherited variants were analyzed because the proband’s mother was also diagnosed with HCM. WGS was performed on the patient’s maternal grandfather and aunt, who have cardiac disease, revealing candidate genetic variants that may contribute to the cardiac phenotype.

**Results:**

Compound heterozygous *MYO19* variants were identified in the neonatal patient. Missense c.203C > G (p.A68G) and frameshift c.275_276del (p.E92Vfs*19) variants were identified, which were located in the myosin motor domain, a functionally crucial region of the *MYO19* protein. Maternally inherited missense variants were identified in *SURF1* and *ETFDH*. All three genes are associated with mitochondrial function, and in silico prediction tools suggest that these variants are likely damaging. Other candidate genetic variants possibly contributing to the cardiac phenotype were also detected in the extended maternal family.

**Conclusions:**

To the best of our knowledge, this study represents the first report proposing *MYO19* as a candidate gene for HCM and highlights the potential role of oligogenic inheritance in the etiology of the disease. Furthermore, plausible candidate variants of other mitochondria-related genes, such as *MYO19*, *SURF1*, and *ETFDH*, were identified, and other family members were investigated to support the pathogenesis of HCM further. Given the limited understanding of the genetics of pediatric HCM, these findings contribute valuable insights into its genetic basis in pediatric patients.

**Supplementary Information:**

The online version contains supplementary material available at 10.1186/s13023-025-03871-5.

## Background

Cardiomyopathy (CMP), a heterogeneous heart muscle disease that affects myocardial structure and function [1], is a leading cause of heart failure, with a strong heritable component displaying variable phenotypic expressivity and penetrance [2]. CMP can be broadly classified in terms of its clinical features, and hypertrophic CMP (HCM) is one of its most common forms. HCM is characterized by left ventricular hypertrophy and diastolic dysfunction, potentially leading to heart failure, sudden cardiac death, and arrhythmias [3, 4]. The age at HCM onset varies widely from infancy to adulthood, with a prevalence of approximately 0.2% in the general adult population [5]. Additionally, HCM can be caused by various inheritance modes, including autosomal-dominant (AD), autosomal-recessive, X-linked, and mitochondrial; however, it primarily follows an AD inheritance pattern [2, 6]. 

Pediatric HCM differs from adult HCM in several ways. Pediatric HCM is more heterogeneous, often involving RASopathy syndromes, inborn errors of metabolism (IEM), and neuromuscular diseases as underlying causes, which can make it particularly fatal. The condition is also extremely rare, with a prevalence of approximately 3 in 100,000, most commonly diagnosed during infancy and preadolescence [7, 8]. In neonates with early-onset HCM, the clinical course progresses rapidly from birth, in contrast to late-onset HCM, which typically presents during prepubertal years [8]. Because of the rapid and massive myocardial hypertrophy observed in infants, HCM often results in severe outcomes, with approximately 30% of infants diagnosed before one year of age requiring heart transplantation or succumbing to the condition [1, 9].

Over the past 2 decades, advances in next-generation sequencing have greatly expanded our understanding of the genetic etiology of HCM, particularly in identifying non-sarcomeric variants associated with HCM [10, 11]. Sarcomeric variants are more common in adult HCM, whereas non-sarcomeric variants are commonly associated with pediatric HCM and have a worse prognosis [12–14]. Infants with pathologic hypertrophy caused by non-sarcomeric variants are often associated with mitochondrial dysfunction or IEM, which markedly increases the risk of death [15]. Moreover, the genetic diversity in HCM suggests that multiple pathogenic variants may contribute to the disease, particularly in cases of oligogenic inheritance or with genetic modifiers. In this context, it has been hypothesized that several genetic variants with modest effects could collectively contribute to the phenotype, potentially being distributed among family members, exhibiting low penetrance, or not strictly co-segregating with the disease [16].

Despite advances in identifying the genetic underpinnings of pediatric HCM, significant gaps remain in our understanding of the role of non-sarcomeric gene variants and the mechanism via which they contribute to the early-onset forms of this disease. Current genetic testing primarily focuses on sarcomeric mutations, leaving non-sarcomeric contributors less explored [17]. Furthermore, the complexity of oligogenic inheritance and genetic modifiers in HCM is not fully elucidated, especially in familial cases with variable phenotypes. This study aims to reveal the genetic architecture in a family with a history of cardiac disease and neonatal HCM, focusing on non-sarcomeric variants. Here we report the case of a neonatal patient with HCM carrying novel compound heterozygous variants of *MYO19*: a paternal missense variant c.203C > G (p.A68G) and a maternal frameshift variant c.275_276del (p.E92Vfs*19). Additionally, candidate variants in other mitochondria-related genes (*SURF1* and *ETFDH*) were identified, and other family members that exhibited mild symptoms were investigated.

## Methods

### Ethical considerations

Written informed consent was obtained from all participants. This study was reviewed and approved by the Samsung Medical Center (SMC) Institutional Review Board (IRB) and Korea National Institute of Health IRB (Approval Number: SMC 2019–10-138–005, 2022–02-07-P-A).

### Case presentation

The proband was a neonatal female and the only child of unrelated Korean parents. She was delivered preterm at 32 weeks and 1 day of gestation via emergency cesarean section owing to severe bradycardia in her 19 year-old mother with HCM. At birth, her Apgar scores were 8 and 9 at 1 min and 5 min, respectively. Her birth weight was 1.82 kg (50–75th percentile), height was 40.5 cm (10–25th percentile), and head circumference was 32 cm (75–90th percentile). She was admitted to the neonatal intensive care unit (NICU) for preterm care and developed respiratory distress syndrome (RDS). The patient received a single dose of surfactant via the Less Invasive Surfactant Administration method, followed by biphasic positive airway pressure ventilation. She experienced intermittent apnea until 35 weeks of corrected gestational age and required positive pressure ventilation for 6 days. At one month of age, echocardiography revealed HCM with moderate systolic dysfunction (ejection fraction [EF], 35.8%; fractional shortening, 14.9%), marked left ventricular outflow tract obstruction (LVOTO) with a peak gradient of 55 mmHg, and grade 2 mitral regurgitation (peak gradient, 90 mmHg). Cardiomegaly was confirmed on chest X-ray, and electrocardiography showed right ventricular hypertrophy (RVH) and left septal hypertrophy. The patient was enrolled in a genetic study because of the family history of HCM. Chromosomal analysis and sonographic examinations of the brain and abdomen revealed no abnormalities. She was treated with propranolol and discharged from the NICU at 38 weeks of corrected age, weighing 2.5 kg. The patient was monitored during outpatient visits.

At seven months of age, she underwent diagnostic catheterization following a magnetic resonance imaging (MRI) to evaluate cardiac function and anatomy, which led to a septal myectomy. MRI confirmed HCM with basal septal inferior wall hypertrophy, aneurysmal change of the apex, mid-ventricular obstructive pattern, mid-to-apex endocardial fibrosis, thrombus in the mid-to-apical subendocardium, pericardial effusion, left pleural effusion, and moderate to severe mitral regurgitation (MR). Cardiac catheterization revealed LVOTO, with a peak gradient of 43 mmHg and severe asymmetric septal hypertrophy. Echocardiography revealed preserved left ventricular function with elevated left ventricular end-diastolic pressure and myocardial bridging of the left anterior descending artery. In the initial echocardiography, the septal thickness was measured at 13.5 mm, and the left ventricular posterior wall thickness (LVPWT) was 3.6 mm. On follow-up echocardiography approximately two years later, the septal thickness had increased to a maximum of 33 mm, and the LVPWT had increased to 6.9 mm. A biopsy indicated interstitial and mild endocardial fibrosis. Despite ongoing follow-up and medication adjustments, worsening LVOTO at 25 months of age required a second septal myectomy. The postoperative complications included bleeding and cardiogenic shock, requiring extracorporeal membrane oxygenation support. The patient died of brain death. The main clinical characteristics in patients with hypertrophic cardiomyopathy are summarized in Table 1.

**Table 1:** Cardiac phenotypes and clinical outcomes in patients with hypertrophic cardiomyopathy

No.	Ethnicity	Age at Dx. (year)	Sex	Cardiac phenotype	Major interventions (medication/surgery/ICD)	Arrhythmia (Type)	Clinical outcome
I-1	East Asian	45	M	HCM (septal 35 mm)	No intervention recorded	Not available (normal)	Alive
II-2	East Asian	14	F	Severe diffuse HCM, Dynamic obstruction	Beta-blocker, Septal myoectomy, ICD at 15 yr	+ (VT)	Alive
II-3	East Asian	12	F	Severe HCM	No intervention*	–	Alive
III-1	East Asian	0.08 (1 month)	F	Severe HCM, Apex aneurysm, Mid-ventricular obstruction	Beta-blocker, septal myoectomy (8 months, 25 months), ICD inserted at 25 months	+ (VA)	Died at 3 years

HCM, Hypertrophic cardiomyopathy; ICD, Implantable cardioverter defibrillator; VT, Ventricular tachycardia; VA, Ventricular arrhythmia

^*^Despite medical advice (beta-blocker prescribed) to initiate treatment for HCM, she declined

The patient’s mother had a history of HCM, which presented at the age of 14 years with dyspnea and ventricular tachycardia, requiring cardioversion and implantable cardioverter defibrillator insertion. Echocardiography revealed severe septal hypertrophy, RVH, systolic anterior motion (SAM) with dynamic left ventricular mid-cavity obstruction, grade 3–4 diastolic dysfunction with increased left ventricular filling pressure, and a LVPWT of 18.4 mm. MRI confirmed HCM with diffuse severe septal hypertrophy (septal thickness of maximum 62 mm), right ventricular involvement and myocardial fibrosis. The patient’s mother was managed with nebivolol and regular follow-ups, delivered the proband at 19 years of age, and underwent septal myectomy at 23 years of age (Table 1). The patient’s father was asymptomatic and did not undergo any evaluations.

### Clinical manifestations in extended family

The patient’s maternal grandfather and aunt were diagnosed with HCM during screening echocardiography after the patient’s mother was diagnosed at the age of 14. The maternal grandfather was diagnosed with HCM at an outside hospital, as documented in the referral letter provided by the patient’s mother. The septal thickness was recorded as 35 mm at the age of 45 during the examination. Although the grandfather, at 54 years of age, is not currently on medication and exhibits almost no symptoms, the exact timing of the diagnosis and detailed examination results are not available in our medical records. The patient’s aunt, at the age of 12, was asymptomatic but was referred to our institution after being suspected of having HCM during a check-up at an outside hospital alongside her sister (patient’s mother). The echocardiography performed at our institution revealed severe septal hypertrophy, systolic anterior motion (SAM) without left ventricular (LV) obstruction, and diastolic dysfunction grade 3 with borderline left atrial (LA) enlargement. For the subsequent two years, the patient was monitored through outpatient follow-ups without any interventions or medication. She remained asymptomatic without treatment until the age of 21 years when she developed severe bradycardia during sedation for medical procedures. Transesophageal echocardiography revealed a 34.2 mm asymmetric septal hypertrophy, normal left ventricular size, an EF of 88%, grade 1 diastolic dysfunction, and mild tricuspid regurgitation with an 18 mmHg pressure gradient. Despite medical advice to initiate treatment for HCM, the aunt declined because of the absence of clinical symptoms. The maternal grandmother had normal echocardiograms and was asymptomatic.

### Whole genome sequencing

After obtaining written informed consent, genomic DNA was obtained from whole blood samples of each participant, and genetic examinations were conducted for further diagnosis. Prior to WGS, chromosomal analysis was performed and revealed a normal female karyotype (46, XX). In addition, a targeted gene panel for HCM-associated genes was conducted, which identified no pathogenic variants. Furthermore, we reviewed a curated list of HCM-related genes from OMIM and PanelApp (Additional file 1), but this analysis also revealed no pathogenic variants. A comprehensive whole genome sequencing (WGS) analysis pipeline was established to identify and annotate candidate genetic variants, including single nucleotide variants (SNVs), indels, copy number variants (CNVs), and structural variants (SVs), using trio-based samples from the patient and both parents. The DRAGEN platform was used for variant calling, and sequence reads were aligned to the GRCh38 human reference genome. Variant Quality Score Recalibration was performed using GATK (v4.2.6.1) to ensure the precision of the variant calls. Gene and variant consequence annotations were conducted using the Variant Effect Predictor (VEP) [18], with insertions and deletions of 50 base pairs or fewer classified as indels. Variant filtering and quality control were conducted using Hail (https://hail.is), followed by VEP annotation for extended-family samples where trio-based analysis was not feasible.

The bioinformatic analysis pipeline for WGS followed a previously described method [19]. Briefly, variants with read depths (DP) below 10 or above 1000 were excluded. Allelic balance (AB) thresholds were used to categorize variants: 0.3–0.7 for heterozygous SNVs, 0.2–0.8 for heterozygous indels, and ≥ 0.95 for homozygous variants. High-confidence de novo, compound heterozygous, and homozygous SNV/Indel variants were identified using the DRAGEN-Hail pipeline. DNVs were filtered out if the genotype quality (GQ) in the proband was ≤ 25 or if the variant was observed in more than 0.1% of individuals from the non-neuro subset of gnomAD v3.1.2 (https://gnomad.broadinstitute.org/). Additional exclusion criteria included a proband AB below 0.3, a parental AB above 0.1, or a proband-to-parental depth ratio below 0.3.

The GATK pipeline and Manta within the DRAGEN software were used to call the CNVs and SVs, respectively. Strict quality control criteria were used to obtain high-quality de novo variants (DNVs) and maternally inherited variants. Additionally, CNV and SV analyses were performed on the samples of the recruited extended family members to identify common variants among family members with HCM symptoms, which were absent in the patient’s father. Subsequently, the variants were manually visualized and filtered using Samplot [20] to select true-positive variants. The population frequencies for CNVs and SVs were obtained from gnomAD SVs v2.1.

### Variant identification, in silico analysis, and variant interpretation

Because no causal variants were identified among the known HCM disease genes, the variants detected from the trio analysis were retrospectively evaluated based on their inheritance patterns: de novo, maternally inherited (heterozygous), and inherited from both parents (homozygous and compound heterozygous). The variants discovered after VEP annotation and variant filtering were analyzed from three perspectives (Additional file 2). First, a trio-based analysis was conducted that included DNVs, compound heterozygous variants, and rare homozygous variants. The second analysis focused on variants inherited from the mother because she also had HCM. Additionally, the analysis also included the patient’s maternal grandfather and aunt, both of whom exhibited HCM-like symptoms, to identify common variants among the four individuals. The pathogenicity and candidate status of the filtered and prioritized variants were assessed using clinical databases, phenotype–genotype analyses, and in silico tools (Additional file 3) [19, 21]. Putative candidate variants were confirmed using an Integrative Genomics Viewer (IGV) and Sanger sequencing. Variant descriptions follow the Human Genome Variation Society (HGVS) nomenclature guidelines.

### Sanger sequencing

To validate the variants identified by whole-genome sequencing, Sanger sequencing was performed using the following primer sets:

- *MYO19* (NM_001163735.1): F 5′-TGACCACATTGCTGGTGAAT-3′, R 5′-TATTTGAGCCCGAGTTTGCT-3′. The two variants in *MYO19* were located in close proximity, allowing validation with a single primer set.
- *SURF1* (NM_003172.4): F 5′-AACGTACGGAAGTTGGCATC-3′, R 5′-ACTGAGCCAGCCCTGTTTTA-3′.
- *ETFDH* (NM_004453.4): F 5′-TGTGCAAAACACAGGGAGAA-3′, R 5′-CCCCCTTCTCTTTCCAGTCT-3′.

### Statistical analyses

Functional enrichment analysis was performed using the g:Profiler toolset, especially the g:GOSt tool, which analyzes individual or multiple gene lists for enriched biological processes and pathways. The three candidate genes (*MYO19*, *SURF1*, and *ETFDH*) were analyzed to identify significant Gene Ontology (GO) terms and pathways. To reduce false positive results, g:GOSt applies the g:SCS (set counts and sizes) correction method for multiple testing and reports the adjusted enrichment *P*-values. Additionally, the GeneMANIA server was used to perform network-based analysis, configured to generate interaction networks and identify enriched biological processes and cellular components linked to the candidate genes. Detailed information on g:Profiler and GeneMANIA can be found in their previous research [22, 23].

## Results

### Genetic findings in the proband

Trio-based analyses revealed de novo, compound heterozygous, and rare homozygous variants (Additional file 2). No DNVs were identified within the coding regions or splice sites; however, one compound heterozygous variant of the *MYO19* gene and 14 rare homozygous variants were detected in the coding region. In silico assessment of these 14 rare homozygous variants indicated that they had Combined Annotation Dependent Depletion (CADD) scores of < 15, with PolyPhen and Sorting Intolerant From Tolerant (SIFT) predictions classifying them as benign or tolerated. Additionally, the American College of Medical Genetics and Genomics (ACMG) classification via Varsome determined that all variants, except one, were benign or likely benign. The variant classified as a variant of uncertain significance (VUS) was annotated as a splice region variant, located five base pairs from the splice acceptor site, and has been reported 234 times in normal populations, predominantly among East Asians, in the gnomAD database. Five true-positive de novo CNV losses were identified, including 3 located in intergenic regions and 2 located in intronic regions of *RIMS1* and *MGAM*. In addition, 5 SVs—two deletions and three duplications—were detected. Among these, 1 duplication was located within an intron of *ASB9*, while the remaining variants did not overlap with any gene regions (Additional file 2).

**Fig. 1:**
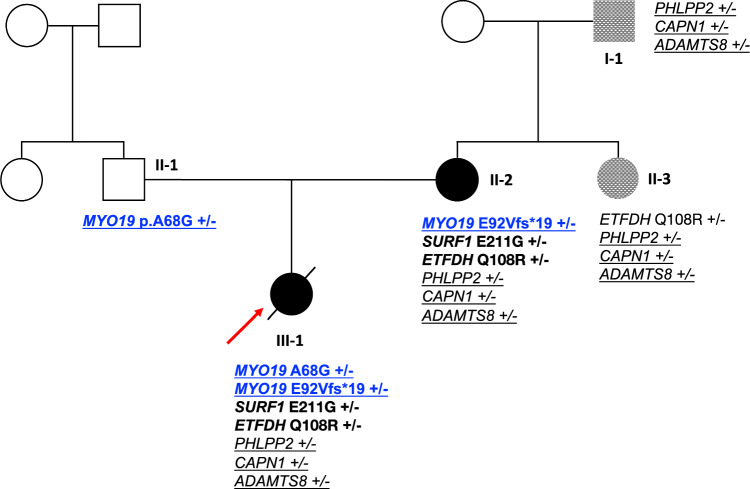
Hypertrophic cardiomyopathy candidate genes based on oligogenic events. Pedigree of the proband’s family. Squares and circles indicate male and female, respectively. Empty symbols denote non-affected individuals, whereas filled symbols denote affected individuals. Checkered marks indicate individuals with mild symptoms. A novel combination in the patient (*MYO19*) is indicated by bold and underbar; bold for maternally inherited variants, and underbar for common variants among the four patients. The proband is shown by the arrow and indicated by a slash (/) because she is deceased

**Fig. 2:**
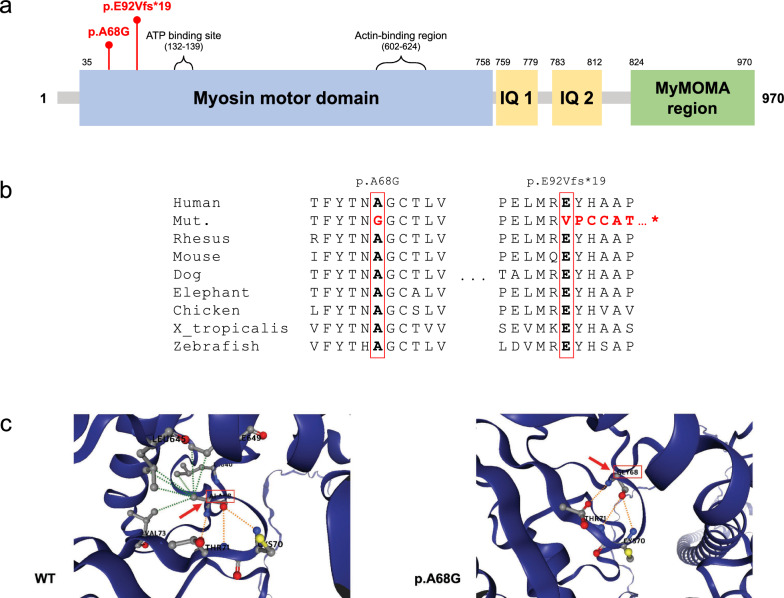
MYO19 protein, amino acid alignment of p.A68G and p.E92Vfs*19, and protein conformational modification prediction. **a** Schematic diagram of the MYO19 protein presenting the functional domains and regions. The p.A68G and p.E92Vfs*19 locations are represented. **b** *MYO19* sequence alignment of the region of the identified variants among representative species. **c** Three-dimensional prediction of the wild-type and mutant protein structures. Because p.E92Vfs*19 was a frameshift variant, only the p.A68G mutant *MYO19* was predicted using DynaMut2 (https://biosig.lab.uq.edu.au/dynamut2/). Red arrows and boxes indicate the region of the variant

As a result of the trio family analysis, 2 promising variants were identified in *MYO19* (NM_001163735.1). These variants were validated using Sanger sequencing (Additional file 4), which confirmed that the proband inherited them through compound heterozygosity. The missense SNV c.203C > G (p.A68G) was inherited from the father, and the frameshift SNV c.275_276del (p.E92Vfs*19) was inherited from the mother (Fig. 1 and Table 2). Both variants are located in exon 5, which encodes a myosin motor domain, a functionally crucial region (Fig. 2a).

**Table 2:** Identified genetic variants in study participants

No.	Sex	Relationship	Genotype						
			*MYO19* (A68G)	*MYO19* (E92Vfs*19)	*SURF1*	*ETFDH*	*PHLPP2*	*CAPN1*	*ADAMTS8*
I-1	M	Maternal grandfather	−	−	−	−	+	+	+
II-1	M	Father	+	−	−	−	−	−	−
II-2	F	Mother	−	+	+	+	+	+	+
II-3	F	Maternal aunt	−	−	−	+	+	+	+
III-1	F	Proband	+	+	+	+	+	+	+

These variants alter highly conserved amino acid residues across different species, including humans, rhesus monkeys, mice, dogs, elephants, chickens, Western-clawed frogs (*X. tropicalis*), and zebrafish (Fig. 2b). Notably, these variants have not been previously reported in PubMed, ClinVar, or Online Mendelian Inheritance in Man (OMIM). In the gnomAD database, the minor allele frequencies (MAF) of the c.203C > G (p.A68G) and c.275_276del (p.E92Vfs*19) variants were < 0.001, with allele counts of nine and one, respectively. According to the ACMG guidelines of VarSome [24], the c.203C > G (p.A68G) variant was classified as a VUS based on PM2, PM3, and BP1, whereas the c.275_276del (p.E92Vfs19) variant was categorized as a VUS based on PM2 (Table 3). Although no in silico prediction results were available for the frameshift variant inherited from the mother, the missense variant from the father was predicted to be damaging and deleterious using CADD, SIFT, and PolyPhen (Table 4).

**Table 3:** List of causative variants in the whole genome sequencing results of the family

Gene	Variant	Type of variant	Inheritance (zygosity)	gnomAD AF (AC)	ACMG classification/criteria	OMIM [MIM No.]	
						Gene	Phenotype
*MYO19*	NM_001163735.1:c.203C > G, p.Ala68Gly	Missense	Paternal (Het.)	0.0000591 (9)	VUS/PM2, BP1, PM3	617379	Not reported
	NM_001163735.1:c.275_276del, p.Glu92ValfsTer19	Frameshift	Maternal (Het.)	0.00000658 (1)	VUS/PM2, PP3	617379	Not reported
*SURF1*	NM_003172.4:c.632A > G, p.Glu211Gly	Missense	Maternal (Het.)	Not found	VUS/PP3, PM2	185620	Charcot-Marie-Tooth disease, type 4K [616684], Mitochondrial complex IV deficiency, nuclear type 1 [220110]
*ETFDH*	NM_004453.4:c.323A > G, p.Gln108Arg	Missense	Maternal (Het.)	0.00001549 (25)	VUS/PP2, PM2	231675	Glutaric acidemia IIC [231680]
*PHLPP2*	NM_015020.3:c.3838C > T, p.Gln1280Ter	Stop gained	Maternal (Het.)	Not found	VUS/PP3, PM2	611066	Not reported
*CAPN1*	NM_005186.4:c.1729 + 1G > A, p.?	Splice donor	Maternal (Het.)	0.00001154 (18)	P/PVS1, PP5, PM2	114220	Spastic paraplegia 76, autosomal recessive [616907]
*ADAMTS8*	NM_007037.6:c.941C > G, p.Ala314Gly	Missense	Maternal (Het.)	0.000007525 (11)	VUS/PM2	605175	Not reported

All variants were validated using Sanger sequencing. Candidate genes and variants are listed in order of priority. ACMG classification was designated by Varsome and manually identified

AF, Allele frequency; AC, Allele count; Het., Heterozygous; ACMG, American College of Medical Genetics and Genomics; VUS, Variant of uncertain significance

**Table 4:** In silico prediction of variants observed in the proband

Gene	Variant	CADD (score/prediction)	SIFT (score/prediction)	PolyPhen-2 (score/prediction)	REVEL (score/prediction)	MutationTaster (score/prediction)	MetaRNN (score/prediction)	AlphaMissense (score/prediction)
*MYO19*	NM_001163735.1:c.203C > G, p.Ala68Gly	27.9/damaging	0.004/deleterious	1.000/probably damaging	0.694/uncertain	1/disease-causing	0.1731/tolerated	0.422/ambiguous
	NM_001163735.1:c.275_276del, p.Glu92ValfsTer19	28.4/damaging	–	–	–	–	–	–
*SURF1*	NM_003172.4:c.632A > G, p.Glu211Gly	26/damaging	0.0/deleterious	0.965/probably damaging	0.912/pathogenic	1/disease-causing	0.9259/damaging	0.584/likely pathogenic
*ETFDH*	NM_004453.4:c.323A > G, p.Gln108Arg	26.1/damaging	0.0/deleterious	0.605/possibly damaging	0.643/uncertain	0.9999/disease-causing	0.7031/damaging	0.11/likely benign
*PHLPP2*	NM_015020.3:c.3838C > T, p.Gln1280Ter	41/damaging	–	–	–	1/disease-causing	–	–
*CAPN1*	NM_005186.4:c.1729 + 1G > A, p.?	34/damaging	–	–	–	1/disease-causing	–	–
*ADAMTS8*	NM_007037.6:c.941C > G, p.Ala314Gly	32/damaging	0.0/deleterious	0.933/probably damaging	0.729/pathogenic	1/disease-causing	0.9504/damaging	0.463/ambiguous

Predictions of the damaging effects of candidate variants as analyzed using the following in silico tools: CADD, SIFT, PolyPhen-2, REVEL, MutationTaster, MetaRNN (referred to in Varsome) and AlphaMissense

Additionally, three-dimensional protein structure prediction analysis supported the hypothesis that the p.A68G variant impacts the stability and function of the MYO19 protein. The substitution of the 68th amino acid from alanine to glycine disrupted hydrophobic interactions with the 40th (leucine, LEU), 73rd (valine), 645th (LEU), and 649th (isoleucine) amino acids (Fig. 2c).

### Maternally inherited variants and functional enrichment analysis

Next, heterozygous variants inherited from the mother were examined. Of the 34,590 maternal variants, 442 were located within coding regions or splice sites. Those with a CADD score of < 20, those predicted as benign by PolyPhen, and those tolerated by SIFT were excluded to prioritize the potentially pathogenic variants, which resulted in the selection of 90 variants. These 90 variants were further classified according to the ACMG guidelines using Varsome; 45 variants were classified as VUS or higher (Additional file 2). Genotype–phenotype associations for these 45 variants were assessed using Automatic Mendelian Literature Evaluation (AMELIE). A manual literature review was conducted for the 21 genes with valid AMELIE outputs (i.e., those without NaN scores), and among them, seven genes with scores ≥ 50 were selected for further investigation. *ETFDH* and *SURF1* were prioritized due to their known roles in mitochondrial function and their relevance to the patient’s clinical features, including respiratory distress syndrome alongside HCM.

We identified 22 CNVs and 75 SVs inherited from the mother. Of the 22 CNVs, 21 were located in intergenic or intronic regions, and 1 CNV loss encompassed an exon of the *GNPAT* gene. *GNPAT* is associated with autosomal recessive Rhizomelic chondrodysplasia punctata, a disorder characterized by craniofacial abnormalities, skeletal dysplasia, and central nervous system involvement. However, due to the low similarity to the patient’s phenotype and the recessive inheritance pattern, this variant was excluded from the list of candidate genes. All SVs were located in intergenic or intronic regions.

Two additional candidate genes—*SURF1* and *ETFDH*—associated with HCM were identified (Table 2). IGV and Sanger sequencing were used to confirm that the proband and her mother carried heterozygous missense variants in the *SURF1* (NM_003172.4:c.632A > G) and *ETFDH* genes (NM_004453.4:c.323A > G), respectively. According to the ACMG guidelines, both variants are classified as VUS. The MAF of these variants in the gnomAD database was non-existent or extremely low. Bioinformatics analysis suggested a potential pathogenic impact on these genes (Tables 3 and 4). According to OMIM, *SURF1* and *ETFDH* are associated with Charcot-Marie-Tooth disease (MIM #616684) and glutaric acidemia IIC (MIM #231680), respectively, with an autosomal recessive inheritance pattern (Table 3). However, no additional pathogenic or likely pathogenic variants were identified in the proband.

**Fig. 3:**
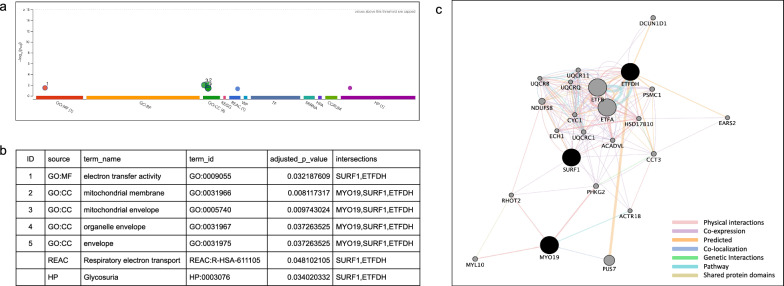
Functional enrichment analysis of *MYO19*, *SURF1*, and *ETFDH* using g:Profiler (**a**, **b**) and GeneMANIA (**c**). **a, b** The x-axis represents the enriched pathways or Gene Ontology (GO) terms, and the y-axis indicates statistical significance (e.g., -log10(*p*-value)). g:SCS (set counts and sizes) correction method was used for multiple testing, and adjusted enrichment P-values were reported. Different colors correspond to various GO categories such as Molecular Function (MF), Biological Process (BP), Cellular Component (CC), or specific pathway databases like the Kyoto Encyclopedia of Genes and Genomes or Reactome. **c** Black nodes represent the three query genes (*MYO19*, *SURF1*, and *ETFDH*), whereas the gray circles indicate additional genes predicted to interact with the query genes. Colored lines indicate different types of interactions. The thickness of the connecting lines represents the strength or confidence of the predicted interactions. Detailed results of the GeneMANIA analysis are provided in Additional file 5

Functional enrichment analysis was performed on 3 genes (*MYO19*, *SURF1*, and *ETFDH*) in the trio family using g:Profiler and GeneMANIA. Functional enrichment analysis of these candidate genes revealed that the mitochondrial membrane and envelope were the top items in the g:Profiler results (*p* = 8.11 × 10^–3^ and 9.74 × 10^–3^, respectively) (Fig. 3a, b). Network analysis using GeneMANIA identified similar gene ontologies, including cellular respiration, electron transport chain, mitochondrial inner membrane, electron transfer activity, and ATP synthesis-coupled electron transport (Fig. 3c and Additional file 5). These results indicate a potentially crucial role for mitochondrial function in HCM onset. Moreover, given that *MYO19*, *ETFDH*, and *SURF1* are all involved in mitochondrial biology, we hypothesize that they may contribute to HCM through an oligogenic mechanism.

### Genetic analysis in extended family

The genomes of the patient and her maternal family were analyzed to determine whether they carried the candidate genes identified in the trio-based analysis. The missense variant in *ETFDH* was identified in the aunt; however, the variants in *MYO19* and *SURF1* inherited from the mother were not shared with the aunt or maternal grandfather (Fig. 1 and Table 2).

Next, the coding region variants common to the four patients with HCM symptoms were identified to identify potential causative variants within the family. Of the 5,258 coding region variants, those with a CADD score of < 20, benign PolyPhen predictions, SIFT-tolerated predictions, and an MAF > 0.001 were excluded. This filtering process resulted in 44 variants: five protein-truncating variants (frameshift, stop gained, and start lost), 10 splice region variants, and 29 missense variants, which were then subjected to in-depth analysis. Based on this analysis, candidate genes were narrowed down to three: a stop-gain variant in *PHLPP2*, a splice donor variant in *CAPN1*, and a missense variant in *ADAMTS8* (Tables 2 and 3). The *PHLPP2* and *ADAMTS8* variants were classified as VUS, whereas the *CAPN1* variant was classified as pathogenic (Table 3). All three variants had a CADD score of ≥ 30. Notably, the SpliceAI score for the *CAPN1* variant was predicted to be 0.84, indicating a loss of the splice donor site (Table 4). No CNVs were common among the four individuals. A total of 277 true-positive SVs shared among the four individuals were identified; however, all were excluded from candidate variants because they were located in intronic or intergenic regions or exhibited high allele frequencies in gnomAD.

## Discussion

This study presents a rare case of a newborn in Korea with novel *MYO19* variants initially diagnosed with prenatal-onset HCM. To the best of our knowledge, this study is the first to propose *MYO19* as a plausible candidate gene for HCM, supporting the involvement of oligogenic inheritance of non-sarcomeric genes in the etiology of the disease. Genetic analysis revealed a novel gene, *MYO19*, that has not been previously associated with HCM. Additionally, two missense variants inherited from the mother were identified in *SURF1* and *ETFDH*. No pathogenic or likely pathogenic variants were detected in sarcomeric genes typically associated with HCM causality. Moreover, neither the proband nor her parents had pathogenic or likely pathogenic variants associated with secondary genetic findings of HCM, including *MYBPC3*, *MYH7*, *TNNT2*, *TNNI3*, *TPM1*, and *MYL3*. Maternal family members with HCM symptoms were also examined, leading to the identification of three additional candidate genes. This study suggests that HCM may occur through oligogenic inheritance, with pathogenic variants accumulating and contributing to varying severity in the manifestation of the disease.

HCM results from autosomal dominant variants of sarcomeric genes; however, variable genetic factors and penetrance often influence it. Notably, cardiac muscle, a highly energy-demanding tissue, demonstrates notable relevance in mitochondrial diseases. Approximately 20–40% of children with mitochondrial disease present with myocardial disorders, with HCM being the most common form of mitochondrial CMP [25], manifesting either with multi-organ involvement or as an isolated feature [26]. Interestingly, all three potential candidate genes identified in this study—*MYO19*, *SURF1*, and *ETFDH—*have been implicated in various mitochondria-related pathological conditions. While mitochondrial diseases typically affect multiple organs, no abnormalities were observed in other organs in this family except for RDS and HCM symptoms. However, mitochondrial CMP should be considered even in the absence of known mitochondrial diseases, as it could be the first or only clinical manifestation.

*MYO19*, which likely has the most significant impact on the patient, is an unconventional myosin localized in the mitochondria [27]. Although its role in cardiac function has not been well studied, previous research has suggested its involvement in breast cancer [28], glioma [29], and hearing impairment [30]. *MYO19* plays a key role in mitochondrial movement and segregation [31] and is essential for the structural organization of mitochondrial cristae. Disruption of *MYO19* function leads to mitochondrial membrane potential changes and reduced oxidative phosphorylation [32]. The MYO19 protein is composed of three domains: motor, neck, and tail. The motor domain is responsible for actin binding, whereas the neck domain contains IQ motifs that interact with calmodulin. The tail region, specifically the MyMOMA domain, is necessary for directing *MYO19* to mitochondria [27, 33].

In this study, 2 novel variants located in *ETFDH* and *SURF1* were identified. *SURF1* encodes the SURF1 protein, which is involved in mitochondrial complex IV assembly. Variants in *SURF1* disrupt cytochrome c oxidase assembly and function, leading to impaired oxidative phosphorylation and mitochondrial dysfunction [34]. The missense variant identified in the proband has not been reported previously and showed a damaging effect on the prediction scores, suggesting a possible contribution to mitochondrial pathology. *ETFDH* encodes electron transfer flavoprotein dehydrogenase, a crucial enzyme involved in mitochondrial fatty acid oxidation and amino acid metabolism. *ETFDH* has been associated with multiple acyl-CoA dehydrogenase deficiencies and mitochondrial disorders [35]. *SURF1* and *ETFDH*-related diseases are typically inherited in an autosomal recessive manner; however, additional pathogenic variants required to develop the disease were not identified in these 2 genes in the patient.

Malfunctions in mitochondrial transfer or structure contribute to the pathogenesis of cardiovascular diseases. Mitochondrial transcellular transfer plays an indispensable role in regulating the development of the cardiovascular system and maintaining normal tissue homeostasis [36]. Mitochondrial transfer from embryonic cardiomyocytes to mesenchymal stem cells is required to initiate stem cell differentiation toward cardiac cells [37]. Furthermore, mitochondrial dysfunction is a pathogenic mechanism in patients with HCM. Mitochondrial fusion and fission impairment induce the accumulation of damaged mitochondria, resulting in severe CMP [38]. Normal hearts possess rich and intact cristae structures in their mitochondria, whereas hypertrophic hearts exhibit severely swollen and deformed mitochondria with blurred cristae structures and membranes [39, 40]. Interestingly, mitochondrial impairment is associated with septal hypertrophy in genotype-negative patients (similar to our proband) with HCM but not in genotype-positive patients [41].

Further analysis, including that of the patient’s maternal family, identified three genes common to all four individuals as candidates for involvement in the oligogenic inheritance model. The stop-gain variant of *PHLPP2* has not been reported in gnomAD and is predicted to be likely pathogenic. Cells overexpressing the *PHLPP2* mutant exhibit marked hypertrophic growth. Additionally, inhibiting *PHLPP2* phosphatase activity increases cardiomyocyte hypertrophy, indicating that *PHLPP2* regulates cardiomyocyte hypertrophy [42]. Calpains are a family of calcium-activated cysteine proteases, and calpain 1 (*CAPN1*) is the predominant isoform in cardiomyocytes, which are involved in signal transduction that leads to myocardial remodeling and heart failure. *CAPN1* is activated by calcium and cleaves several proteins crucial for normal heart function [43]. The splice variant discovered in this family is expected to affect cardiac function by skipping exon 16, resulting in the loss of a key part of the EF-hand domain. Finally, the *ADAMTS8* expression level was markedly increased in patients with dilated CMP [44]. Other members of the ADAMTS family are also implicated in cardiovascular diseases that affect the overall myocardium, cardiac development, and blood pressure [45].

The analysis of VUS is crucial for the advancement of precision medicine. This study suggests that traditional approaches for identifying the causal genes of HCM may not effectively identify the subtle effects of oligogenic inheritance or genetic modifiers. Moreover, multiple genetic variants may account for the varying severity of heart defects observed in individuals with shared variants. However, this study has limitations that need to be addressed in the future. Additional functional analyses are required to validate the effects of the candidate genetic variants identified in this study and experimental evidence and cases from independent families with variants in *MYO19* are essential to establish *MYO19* as a novel gene associated with HCM.

## Conclusion

Compound heterozygous variants in *MYO19* and several candidate variants in 5 other genes were identified using an HCM family analysis. The present case is particularly noteworthy, as the patient died at a young age, whereas maternal family members with the same disease exhibited relatively milder symptoms. Therefore, the *MYO19* gene was prioritized, which possibly acted in a biallelic manner, potentially contributing to the more severe phenotype in the patient. However, because the maternally inherited genes (*SURF1* and *ETFDH*) were associated with mitochondrial morphology or function in the network analysis and were linked to each other, the possibility that the genetic variants in these genes acted as modifiers or part of an oligogenic inheritance pattern cannot be excluded.

To the best of our knowledge, this is the first reported disease-causing variant of the *MYO19* gene associated with neonatal HCM. This study provides valuable insights into the multigenic effects of disease-related genes on neonatal HCM. However, further research and experimental evidence are warranted to elucidate the precise mechanisms by which these genes contribute to the complex phenotypes in HCM and mitochondrial CMP.

## Supplementary Information

Additional file 1.

Additional file 2. 

Additional file 3.

Additional file 4.

Additional file 5.

## Data Availability

All data generated or analyzed during this study are included in this published article and its supplementary information files.

## Abbreviations

ACMG: American College of Medical Genetics and Genomics
AD: Autosomal-dominant
CADD: Combined annotation dependent depletion
CMP: Cardiomyopathy
CNV: Copy number variant
EF: Ejection fraction
HCM: Hypertrophic cardiomyopathy
IEM: Inborn errors of metabolism
IGV: integrative genomics viewer
LEU: Leucine
LVOTO: Left ventricular outflow tract obstruction
MAF: Minor allele frequency
MRI: Magnetic resonance imaging
NICU: Neonatal intensive care unit
OMIM: Online mendelian inheritance in man
RDS: Respiratory distress syndrome
RVH: Right ventricular hypertrophy
SIFT: Sorting intolerant from tolerant
SNV: Single nucleotide variant
SV: Structural variant
VEP: Variant effect predictor
VUS: Variant of uncertain significance
WGS: Whole genome sequencing
