# Supplementary figures and images for: Identification of novel MYO19 variants in neonatal hypertrophic cardiomyopathy: a familial analysis revealing oligogenic contributors to disease severity
Source: Orphanet J Rare Dis. 2025 Jul 9;20:349. doi: 10.1186/s13023-025-03871-5 (PMC12239423; doi:10.1186/s13023-025-03871-5)

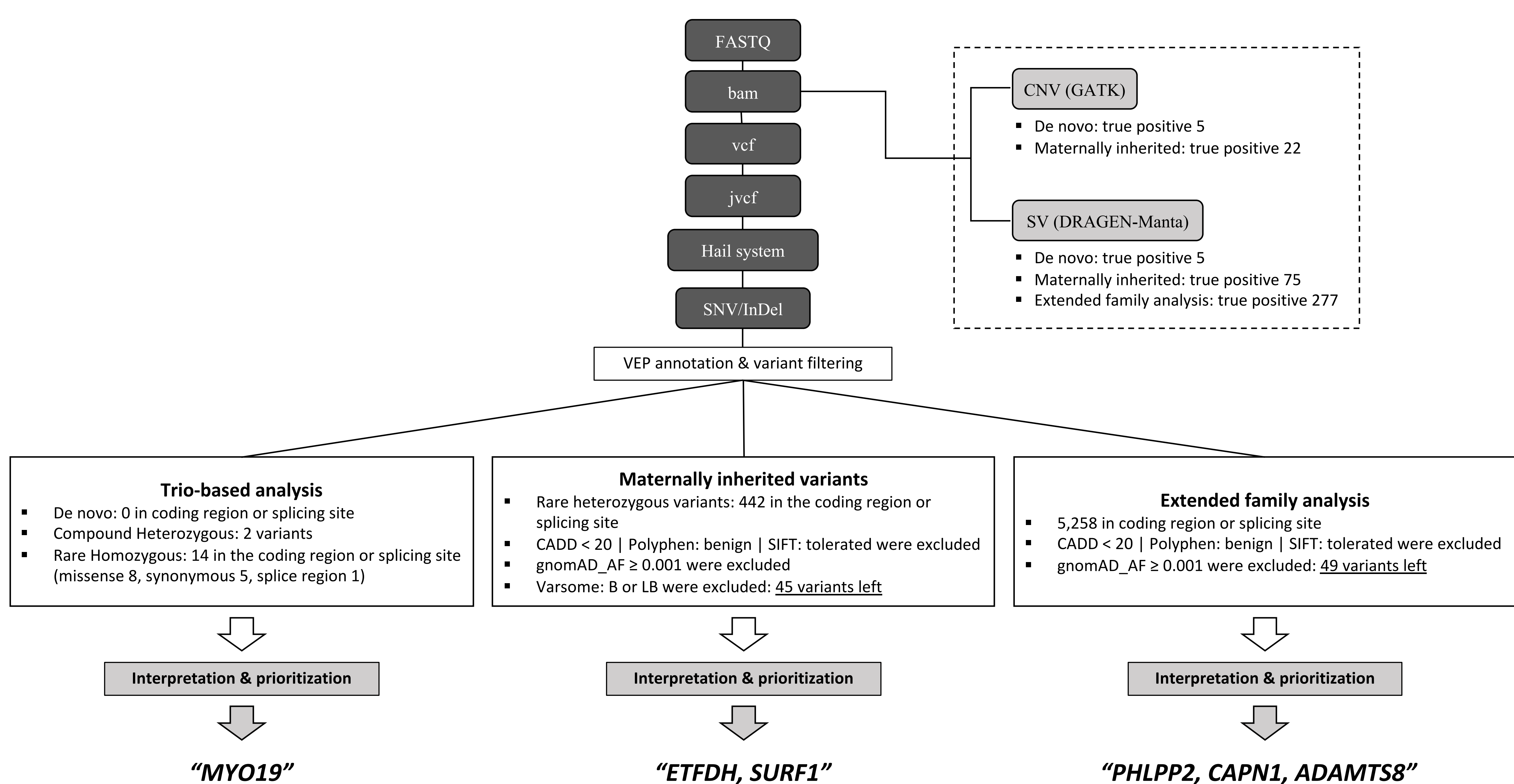

Supplement: Supplementary file 2 — Additional file 2. [file 13023_2025_3871_MOESM2_ESM.pdf]
