# Supplementary material for: Identification of novel MYO19 variants in neonatal hypertrophic cardiomyopathy: a familial analysis revealing oligogenic contributors to disease severity
Source: Orphanet J Rare Dis. 2025 Jul 9;20:349. doi: 10.1186/s13023-025-03871-5 (PMC12239423; doi:10.1186/s13023-025-03871-5)
Supplement: Supplementary file 4 — Additional file 4. [file 13023_2025_3871_MOESM4_ESM.pdf]

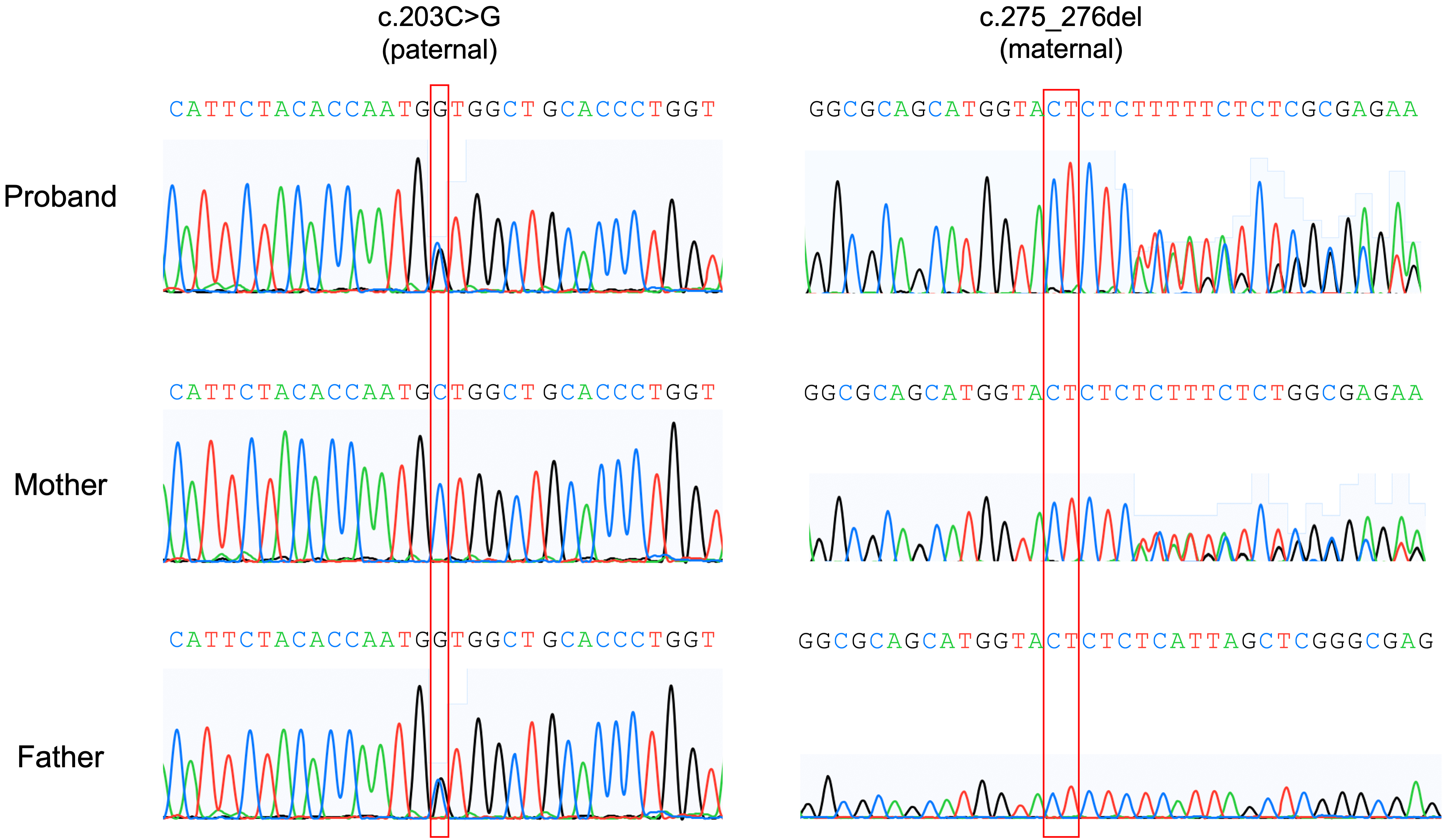
c.203C>G
(paternal)
c.275_276del
(maternal)
Proband
CATTCTACACCAATGGTGGCT GCACCCTGGT
GGCGCAGCATGGTACTCTCTTTTTCTCTCGCGAGAA
Mother
CATTCTACACCAATGCTGGCT GCACCCTGGT
GGCGCAGCATGGTACTCTCTCTTTTCTCTGGCGAGAA
Father
CATTCTACACCAATGGTGGCT GCACCCTGGT
GGCGCAGCATGGTACTCTCTCATTAGCTCGGGCGAG
